# Second Reading Passed on Friday, March 28: The Debate

**Published:** 1919-04-05

**Authors:** 


### NURSES' REGISTRATION BILL.

### Second Reading Passed on Friday, March 28: The Debate.

**Major Barnett,** having moved "That the Bill be now read a second time," said in the course of his speech: At present there is State registration in South Africa not only in Cape Colony, but in Natal, the Transvaal, and the Orange River Colony. In Canada the Provinces of Ontario, Manitoba, Alberta, and British Columbia have all got State registration of nurses. In Australia Queensland has adopted the principle, and in India the Bombay Presidency. The principle is in force in forty-six of the United States of America. It was in force in the German Empire and Belgium. There is nothing whatever in our Statute Book to define a nurse's position or qualifications, and it is in order to give nursing the dignity of a profession that this Bill is introduced. I ought perhaps to say something of the Central Committee which is responsible for drawing up this measure. It represents no fewer than 30,000 medical practitioners and nurses.

The General Nursing Council.

The first thing we propose to establish is a General Nurses' Council. The Crown must be represented by persons appointed by the Privy Council. A certain number of medical practitioners will be nominated by the Local Government Board—it will be the Ministry of Health if certain legislation now before the House goes through—and by the British Medical Association. Then we come to the most important part of the Council—the elected part. The part which will be elected by registered trained nurses will consist partly of matrons and partly of trained nurses. There is provision also for representation of the nurses' training schools. It is quite clear that there ought to be representation on the General Council of Nurses for the lay element, boards of governors, and others who are interested in this question. It is quite obvious that the General Council cannot be created at once, because it depends on having an electorate, and that electorate can only be created through the Register. Therefore this Bill provides for what I may call a provisional or initial council which will hold office for two years and which will draw up rules and regulations to govern the profession of nursing. It will be the business of the initial council to hold office for two years and to devote itself to the formation of the register.

Then there is a provision for the election of direct representatives when the Register has been formed. The rules which are made by the Nursing Council are not to have effect until they are approved by the Privy Council, and it is only with that approval that they obtain the force of law. There is also a provision setting up divisional boards for England, and Scotland, and Wales, and Ireland. There is an appeal from those divisional boards to the Council, and that ought to be a safeguard against any possibility of oppression.

Three Years' Period of Grace.

The House will, I am sure, be anxious to know what is to happen to those people who are nurses already. In the first place, there is what I may call a period of grace, or an interregnum, of three years during which any person who is *bonâ fide* carrying on the business or profession of a nurse will be entitled to come forward and claim registration.

The second Sub-section of that Clause provides for registration, provided the person holds a certificate as to training from the Lords Commissioners of the Admiralty, or as a nurse authorised by the Army Council for soldiers of the Royal Army Medical Corps, and the third Sub-section if the person holds a certificate from the Local Government Board for Ireland or from the Local Government Board for Scotland as to qualifications, and by the fourth Subsection if the person produces evidence satisfactory to the Council of training prescribed by the rules framed under the provisions of this Act and has, in addition, been for at least three years in *bonâ fide* practice as a nurse. After the period of three years no one can be registered except those who satisfy the requirements, which are that the person who claims to be registered shall be a British subject of at least twenty-one years of age and of good character, and has gone through the curriculum laid down by the Council of Nursing, which will include an examination.

Supplementary Registers.

There is to be a supplementary Register of male nurses, and a further supplementary Register of mental nurses, and the House will see that both of those supplementary Registers are of a type of nurses who are really not sick nurses at all. Male nurses and mental or psychological nurses have a different training altogether, and will be on a supplementary Register. A question will, no doubt, be raised in Committee, if the Bill secures its second reading, whether there ought not to be further supplementary Registers for the profession of children's nurses, for instance. I do not wish to shut the door to any class of nurses who are carrying on the profession at present.

The Financial Aspect.

I come to a point on which there has been a good deal of criticism of this Bill, and it is that of finance. A document has been circulated to hon. members which endeavours to riddle this Bill with criticism on the ground that we do not provide proper financial means for carrying out its provisions. At the present time it is estimated that there are 80,000 practising nurses in the United Kingdom, and it is estimated that if this Bill goes on the Statute Book 50,000 nurses will register in the first year at a fee that is not prescribed yet, but which is suggested at two guineas. That will give 100,000 guineas, which, invested in the direction which my right hon. friend will be able to indicate to us, will give 5,000 guineas of annual income with absolute safety. That will be quite sufficient, with the new fees coming in, and the new nurses registering in each year, and with the examination fees, which it is suggested should be something from one to three guineas—and I favour the higher figure—to carry out all the purposes of this Act. It is suggested in the leaflet that has been circulated that our Bill fails entirely to provide any financial basis adequate to sustain the expenses of conducting examinations, of publishing annually the Register, of erecting or running buildings suitable for central offices, of paying examiners, travelling expenses, etc. I will say at once that those who are responsible for the preparation of this Bill are not suffering from megalomania. They have no desire to spend large sums of money in bricks and mortar, to erect enormous offices where the business of this General Council of Nursing shall be carried on. That is not their programme. I have indicated a source from which 100,000 guineas can reasonably be expected to come in the first year of the operation of the Act, and year by year, as other nurses come forward, there will be a fund for keeping up the Register, while the examination fees will provide money for the proper training of examiners and the holding of the examinations. People say that nurses will not pay as much as five guineas for professional training and registration. Midwives pay between £20 and £30 for their training and registration, and I do not think that any nurse or her people will object to paying two guineas once for all for the privilege of registration, and an examination fee at the highest of three guineas, making five guineas in all.

Danger of Losing the Best Nurse.

**Mr. Briant** said that most of his life having been spent amongst those to whom nursing is of vital and first importance, he was more and more convinced that, unless some step were taken to give nurses the status which they have a right to demand, and which they have deserved, then in the future, when nurses will be required more than ever before, it will be found there is a scarcity. Unless the nursing profession has some security that it will have a definite status in the nation, and be protected from being possibly misunderstood and misrepresented as belonging to that large class of people who pose as nurses without any efficiency and without any real training, then we shall lose that better class of nurse which is so important, and indeed essential, for the health of the nation.

PLEA FOR A STATE GRANT.

**Sir Donald Maclean,** in supporting the second reading, drew attention to Clause 12 dealing with the status of and the provision for existing nurses. There were some very earnest and zealous medical reformers who held that the training of nurses should not be less than three years. In the London Hospital, where he understood the training was about two years, a more skilled and zealous body of nurses was not to be found in the United Kingdom. If under this clause adequate protection were not given it might affect adversely the person holding a certificate who had been trained for such a period at a hospital approved by the Council. The greatest possible care should be exercised in the provision of adequate safeguards. They ought to have State assistance. A grant of £2,000 a year would be one of the most reproductive expenditures the State could undertake in the training and development of this splendid class of women.

**Major Sir Samuel Scott,** alluding to the document put forth by the College of Nursing as a first-rate document in support and not against the Bill now before the House, assured the Government that the promoters of the Bill would be only too ready to make it as good a Bill in Committee as they possibly could by accepting any reasonable amendment.

GENERAL COUNCIL CRITICISED.

**Mr. Lyle** addressed the House for the first time, and said there were certain clauses in the Bill which he hoped would be amended in Committee. After studying the question for a very long time, he found that what convinced him most was, not the literature issued by the College of Nursing, but the literature issued by the promoters of the present Bill. With regard to the proportion of nurses on the General Nursing Council, which is the principal point of the whole Bill, and of which there are to be forty-one members, he thought the number of nurses given to England was altogether out of proportion. There are to be eighteen nurses on that Council, and there are eight given to England and four to Ireland. Anybody who knew anything about the respective populations and about the number of nurses in hospitals and so on in the two countries would realise that that proportion was utterly unsound. Then again, out of the eight English nurses four must be matrons. He had the greatest respect for matrons, but this was called a democratic Bill. It was the same all the way through. Out of four Scottish nurses two must be matrons, and out of four Irish nurses, again, two must be matrons. There was another very serious point. The managers of the voluntary hospitals and Poor-Law infirmaries had only four representatives out of forty-one on this Council; for the whole of England and Wales there are only two.

Whilst this General Council of forty-one was open to these criticisms, the Provisional Council which was to be set up temporarily, pending the appointment of this other body, was infinitely more to be criticised. On that Provisional Council there were to be twenty-eight members, and of eighteen nurses no less than twelve were given to the conglomeration of societies mentioned by the promoters of the Bill. These societies were taking to themselves twelve out of the eighteen nurses, and only two were to be given to the College of Nursing, which represented between 12,000 and 13,000 nurses. He did not think it fair that out of eighteen nurses on the Provisional Council twelve should be the nominees of the Central Committee for the State Registration of Nurses and only two of the College of Nursing. Then, again, there was not a single representative of any of the managing committees of the voluntary hospitals or Poor-Law infirmaries.

**Lieutenant-Colonel Raw** gave strong support to the principle embodied in this Bill. The great advances in surgery and medicine had made it necessary that a nurse should be thoroughly trained. She ought to be placed on a State register and under the protection of the authority of Parliament.

LABOUR PARTY SUPPORT.

**Mr. F. Roberts,** in a maiden speech, on behalf of the Labour Party, pleaded that there should not be sweated conditions in any industry, and least of all should there be sweated labour in the noble profession of nursing. He pleaded, too, that the nurses should be given a larger measure of control of the conditions under which they were called upon to work. The Labour Party would ask that the most generous treatment financially which could be given should be undertaken by the State.

**Sir Watson Cheyne** suggested that the Government ought to take this Bill under its own ægis, and the natural Department of the Government to do so was the coming Ministry of Health.

**Mr. Rawlinson** declared he had never believed in State registration for nurses. He thought we wanted an official directory of nurses, on the lines of Lord Balfour of Burleigh's Bill in the House of Lords, and in that directory there should appear the qualifications and training of the nurses and any other necessary particulars. We wanted also the power for any training school or hospital to copyright its nursing uniform.

**Colonel Wedgwood** contended that the Bill would have the effect of narrowing the field of supply of nurses and the avenues by which women approached the profession. He did not propose to offer opposition to the Bill.

GOVERNMENT ATTITUDE.

**Major Astor** (Parliamentary Secretary to the Local Government Board) said: Yesterday afternoon, in Standing Committee A upstairs, when the Health Bill was being considered, an amendment was put forward dealing with the registration of nurses. I was obliged to point out to the proposer that it was quite impossible in one clause to deal with such a very difficult question as the registration of nurses. The fact that we have before us this afternoon a Bill of twenty-six clauses dealing with the same thing shows that we were wise in objecting to that particular proposal yesterday. I am sure that anything which improves the status and efficiency of the nursing profession must tend towards the success of the Ministry of Health. The discussion which we have had shows that there is an overwhelming support among members of the House in favour of the principle of setting up a Statutory Register for nurses. That is the main underlying principle of the Bill. There is a great deal of interest and support outside. We have to-day what is normally the public gallery of this House practically a ladies' gallery.

As regards the attitude of the Government, I do not propose to speak at any length or to examine in detail the proposals set out in the Bill. All I want to say is we are generally in favour of the principle contained in the Bill. Hon. Members probably know there is another Bill, the backers of which have not been so successful in respect to their Bill. Because of that their proposals are not now before the House. I feel certain that the points on which that other Bill differs from this particular Bill will be considered by the Standing Committee when this Bill is upstairs. I will only to-day suggest to the House that we should now let this Bill be read a second time. It will then go upstairs, be examined, and be subjected to criticism and amendment. When it returns to this House, if it is in such a shape that the Government can give it their approval and support, well and good. I can only repeat what I said just now, that we are in favour of the principle. Of course, the attitude which the Government will take up when the Bill comes down here must of necessity depend upon its shape then. We welcome any measure to improve the status of the nursing profession. It has won the admiration of the world as a whole, and during the last few years, during these trying years of war, it has earned the undying gratitude of millions of people.

The Bill was accordingly read a second time and committed to a Standing Committee.

